# New Inhibitors of Laccase and Tyrosinase by Examination of Cross-Inhibition between Copper-Containing Enzymes

**DOI:** 10.3390/ijms222413661

**Published:** 2021-12-20

**Authors:** Dinesh Chaudhary, Fangchen Chong, Trilok Neupane, Joonhyeok Choi, Jun-Goo Jee

**Affiliations:** Research Institute of Pharmaceutical Sciences, College of Pharmacy, Kyungpook National University, 80 Daehak-ro, Buk-gu, Daegu 41566, Korea; pudinesh26@gmail.com (D.C.); chongfangchen@foxmail.com (F.C.); ozitrilok99@gmail.com (T.N.); crowz124@naver.com (J.C.)

**Keywords:** cheminformatics, ceruloplasmin, dopamine-β-hydroxylase, laccase, tyrosinase

## Abstract

Coppers play crucial roles in the maintenance homeostasis in living species. Approximately 20 enzyme families of eukaryotes and prokaryotes are known to utilize copper atoms for catalytic activities. However, small-molecule inhibitors directly targeting catalytic centers are rare, except for those that act against tyrosinase and dopamine-β-hydroxylase (DBH). This study tested whether known tyrosinase inhibitors can inhibit the copper-containing enzymes, ceruloplasmin, DBH, and laccase. While most small molecules minimally reduced the activities of ceruloplasmin and DBH, aside from known inhibitors, 5 of 28 tested molecules significantly inhibited the function of laccase, with the K_i_ values in the range of 15 to 48 µM. Enzyme inhibitory kinetics classified the molecules as competitive inhibitors, whereas differential scanning fluorimetry and fluorescence quenching supported direct bindings. To the best of our knowledge, this is the first report on organic small-molecule inhibitors for laccase. Comparison of tyrosinase and DBH inhibitors using cheminformatics predicted that the presence of thione moiety would suffice to inhibit tyrosinase. Enzyme assays confirmed this prediction, leading to the discovery of two new dual tyrosinase and DBH inhibitors.

## 1. Introduction

One third of the total enzymes in living organisms include metals to maintain their structures or implement their functions [1,2]. The most common metalloenzymes are zinc-containing proteins, followed by those containing iron. Malfunction of metalloenzymes often leads to diseases, making metalloenzymes a potential target for drug discovery. However, inhibitors of metalloenzymes have been less developed by drug researchers. Thus far, FDA-approved drugs include fewer than 70 metalloenzyme inhibitors [1,3], most of which are inhibitors of four types of proteins: lanosterol 14α-demethylase, carbonic anhydrase, histone deacetylase, and metalloprotease [3]. This limited number may reflect the difficulty caused by the nonspecificity of metal-binding groups (MBGs) in small molecules [4,5].

Copper-containing enzymes account for <5% of all metalloenzymes [3,6,7,8]. The cellular level of copper is much lower than that of zinc, and copper-containing proteins are much fewer in number than zinc-containing proteins. The small molecules that inhibit copper-containing enzymes are scarce, except for those that inhibit tyrosinase and dopamine-β-hydroxylase (DBH) [9,10,11,12,13]. The FDA has approved hydroquinone as a depigmenting agent through the inhibition of tyrosinase [3]. No other drug targeting copper-containing enzymes has been approved. In addition to tyrosinase and DBH, copper-containing enzymes include ceruloplasmin, lysyl oxidase, peptidylglycine monooxygenase, bilirubin oxidase, galactose oxidase, hexose oxidase, ascorbate oxidase, nitrite reductase, nitrous-oxide reductase, quercetin 2,3-dioxygenase, and laccase.

Several studies have reported on the selectiveness of metalloenzyme inhibitors across metals, exhibiting off-target effects [2]. However, information on off-target effects in copper-containing enzymes is limited. We previously reported new types of tyrosinase inhibitors by computation and experimentation [14,15,16,17]. In this study, we extend our knowledge on the selectiveness of copper-containing enzyme inhibitors by determining whether tyrosinase inhibitors could inhibit DBH and ceruloplasmin in humans and laccase in fungus. Most metalloenzyme inhibitors contain a MBG that directly interacts with the catalytic metals. The type of MBG necessary to contact specific catalytic metals relies on the character of the metals and the geometry around the metal-containing region. Geometry differs in each enzyme; however, the atomic properties interacting with metals may persist between inhibitors for copper-containing enzymes. Therefore, we hypothesized that the probability of finding new inhibitors will increase more by checking cross-inhibition than random screening. We selected these copper-containing enzymes considering the commercial availability of proteins and the feasibility of in-house enzyme assays.

Tyrosinase is a key enzyme that helps in melanin production in living organisms [18,19]. It catalyzes the conversion of tyrosine, the substrate for melanin biosynthesis, into dihydroxyphenylalanine (DOPA) and subsequently into dopaquinone, which is then spontaneously converted into melanin. The protein possesses evolutionarily conserved six histidines and two juxtaposed copper ions as metal cofactors. Three histidines form coordinate bonds with a copper ion.

Ceruloplasmin is a ferroxidase enzyme that carries a significant portion of copper in plasma and is mainly present in hepatocytes. It consists of six copper atoms, forming a trinuclear cluster that serves as the binding site for oxygen molecules in the catalytic process [20,21]. Ceruloplasmin catalyzes oxidation of Fe^2+^ into Fe^3+^_,_ which is associated with transferrin. Mutation of ceruloplasmin results in high iron contents in the retina, pancreas, liver, and brain, resulting in a condition known as aceruloplasminemia. Ceruloplasmin overexpression is associated with various neoplastic and inflammatory conditions [21,22,23].

DBH is a monooxygenase that primarily catalyzes the conversion of L-DOPA to norepinephrine, which is critical for regulating neurotransmission. DBH is widely expressed in the chromaffin cells of adrenal glands and the neurosecretory vesicles of central and peripheral neurons. Its involvement in many neuronal abnormalities, such as Alzheimer’s disease, anxiety, and Parkinson’s cocaine resistance has been suggested [24,25]. DBH contains two copper-containing domains: CuH and CuM. Three histidines form a coordinated cluster with a copper atom in CuH, and CuM contains two histidines and one methionine, which coordinate with a copper atom [25,26].

Laccase is an enzyme that catalyzes the oxidization of polyphenol. It is widely distributed in bacteria, fungi, insects, and higher plants [27]. The catalytic center of laccase contains two evolutionarily conserved clusters that are composed of one (T1 site) and three (T2 and T3 sites) copper atoms, respectively. Industry has utilized high laccase reactivity in various fields, such as wastewater treatment, drug analysis, ethanol production, wine clarification, delignification, and bioremediation, with an emphasis on decolorizing dyes [28,29].

Our experiments quantified the cross-inhibitory activities of known tyrosinase inhibitors against the ceruloplasmin, DBH, and laccase using biochemical assays and orthogonal biophysical methods. Prediction through cheminformatics with tyrosinase and DBH inhibitors and experimental tests also resulted in new dual tyrosinase and DBH inhibitors.

## 2. Results

### 2.1. No New Inhibitor of Ceruloplasmin and Dopamine-β-Hydroxylase Was Identified by Enzyme-Based Assays with Tyrosinase Inhibitors

**Figure 1:**
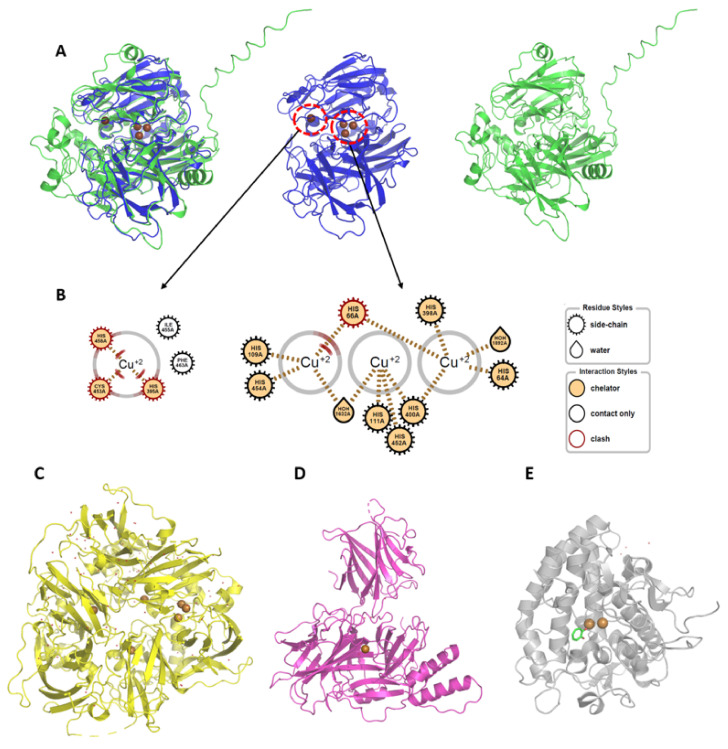
Structures of copper-containing metalloenzymes in this study. (**A**) Overlaid laccase structures. Structures of T. versicolor (blue) and A. oryzae (green) laccases are overlaid. The PDB code for T. versicolor (center) is 1GYC, and the UniProt code for A. oryzae is I8U4Z0. AlphaFold [31] generated the structure for A. oryzae (right). Copper atoms are represented by brown spheres. (**B**) Catalytic metal-containing regions. The 2D diagram was prepared with Openeye Grapheme Toolkit. Two copper clusters (T1 (left), T2, and T3 (middle) sites) in T. versicolor are drawn with definition (right). (**C**) Ceruloplasmin, (**D**) dopamine-β-hydroxylase (DBH), (**E**) tyrosinase. The used PDB codes for ceruloplasmin, DBH, and mushroom tyrosinase are 4ENZ, 4ZEL, and 2Y9X, respectively. The brown spheres indicate copper atoms. Tropolone complexed in tyrosinase is shown with sticks.

We selected five copper-containing enzymes from the four types of enzyme families, considering their availabilities. These enzymes are DBH and ceruloplasmin from humans, tyrosinase from mushrooms, and two laccases from fungi. The number of copper atoms contained in tyrosinase, laccase, DBH, and ceruloplasmin are 2, 4 (3 + 1, a cluster with three coppers and one copper), 2, and 6 (3 + 2 + 1), respectively. The three-dimensional (3D) structures in the four types of enzymes are entirely different from each other, and their catalytic centers are not overlaid [30] (Figure 1). The two laccases from Trametes versicolor and Aspergillus oryzae have limited sequence identity, with a value of 26.8% (Figure S1). However, the residues for chelating coppers are well conserved, indicating that the 3D structures of the catalytic sites in the laccases are similar [7] (Figure S1).

**Figure 2:**
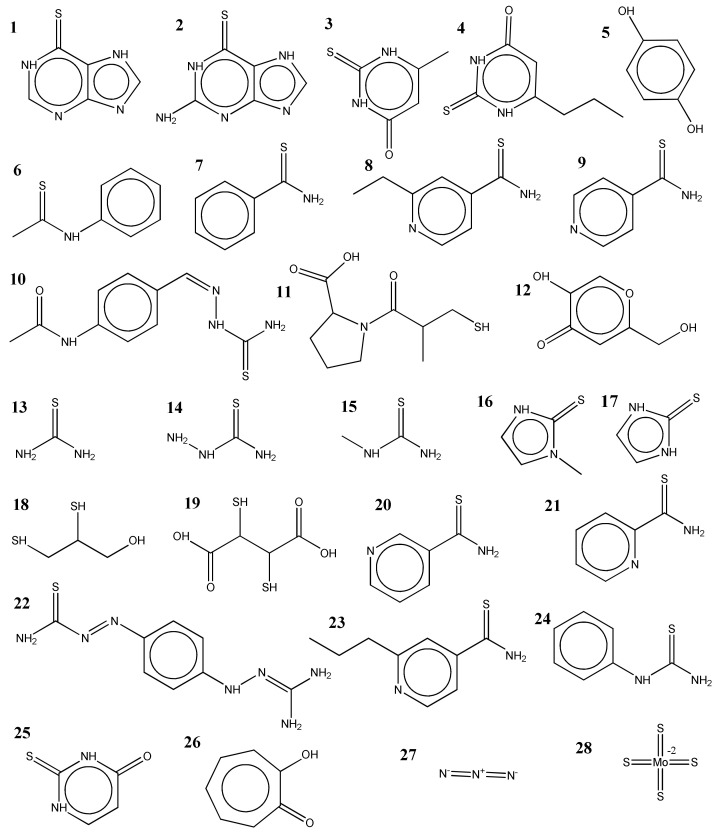
The molecules tested in this study were as follows: **1**. mercaptopurine, **2**. thioguanine, **3**. methylthiouracil, **4**. propylthiouracil, **5**. hydroquinone, **6**. thioacetanilide, **7**. thiobenzamide, **8**. ethionamide, **9**. thioisonicotinamide, **10**. thioacetazone, **11**. captopril, **12**. kojic acid, **13**. thiourea, **14**. thiosemicarbazide, **15**. N-methylthiourea, **16**. methimazole, **17**. mercaptoimidazole, **18**. dimercaptopropanol, **19**. dimercaptosuccinate, **20**. pyridine-3-carbothioamide, **21**. pyridine-2-carbothioamdie, **22**. ambazone, **23**. prothionamide, **24**. phenylthiourea (PTU), **25**. thiouracil, **26**. tropolone, **27**. sodium azide, and **28**. ammonium tetrathiomolybdate (ATMD).

The ChEMBL database for small bioactive molecules contains several hundred and tens of tyrosinase and DBH inhibitors, respectively [32,33]. However, no inhibitor of ceruloplasmin exists, and only an inorganic compound, sodium azide, is registered as a laccase inhibitor. For the tyrosinase inhibitors in this study, we first selected 19 molecules based on our previous studies: prothionamide, thioguanine, mercaptopurine, methimazole, thioacetanilide, thioisonicotinamide, thiouracil, methylthiouracil, propylthiouracil, thiourea, N-methyl thiourea, thiosemicarbazide, ethionamide, pyridine-2-carbothioamide, pyridine-3-carbothioamide, ambazone, thioacetazone, thiobenzamide, and hydroquinone [15,16,17]. These molecules include drugs that have been repurposed by the combined use of computational, biochemical, and biophysical methods. We included captopril and mercaptoimidazole according to other studies. Three molecules, i.e., phenylthiourea (PTU), kojic acid, and tropolone, were included because they have been widely used as control inhibitors. Two inorganic molecules, sodium azide and ammonium tetrathiomolybdate (ATMD), were also used for comparison. The tyrosinase inhibitors largely comprise molecules that possess a poly hydroxyl moiety and thione [15,16]. Our test molecules cover these two classes (Figure 2 and Figure S2).

Dimercaptosuccinate and captopril are inhibitors of tyrosinase. Dimercaptosuccinate, which was selected owing to its chemical similarity to dimercaptopropanol, inhibits tyrosinase activity, with an inhibitory constant (*K_i_*) of 7.6 µM (Figure S3). No studies have reported dimercaptosuccinate as a tyrosinase inhibitor. Day and Cohen showed that captopril slightly inhibited the function of tyrosinase [2], which is inconsistent with another study that reported captopril as an inhibitor [34,35]. Our results confirm that captopril inhibits tyrosinase activity (Figure S3). 

No molecule significantly inhibited ceruloplasmin activity at a concentration of 50 µM, except for sodium azide and ATMD (Figure S4). ATMD and tropolone significantly decreased DBH activity, whereas sodium azide and dimercaptopropanol showed moderate inhibition (Figure S5). Our data are consistent with the report that tropolone is an inhibitor of DBH [36]. 

### 2.2. Enzyme-Based Assays Identified New Organic Laccase Inhibitors 

**Figure 3:**
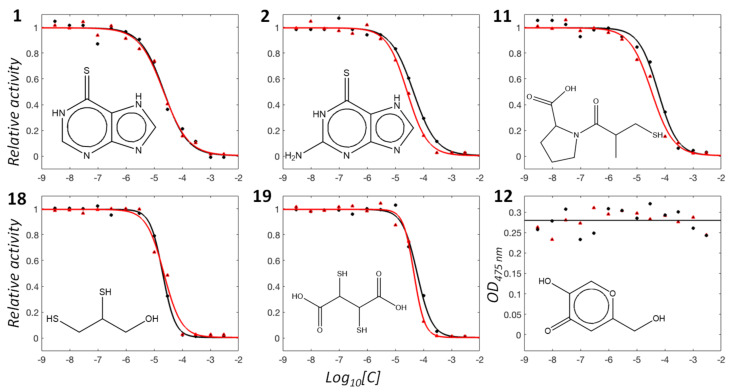
Representative graphs showing concentration-dependent inhibition profiles of test molecules for *T. versicolor* (black) and *A. oryzae* (red) laccases. The activities are scaled to have a relative value of 0–1 for **1**. mercaptopurine, **2**. thioguanine, **11**. captopril, **18**. dimercaptopropanol, **19**. dimercaptosuccinate, and **12**. kojic acid. The enzyme activity without inhibitor is adjusted as 1 for **1**−**11**. For **12**, the raw values were included for clarity.

Five organic and two inorganic molecules showed substantial inhibitory activities against laccases at a concentration of 50 μM. These molecules are mercaptopurine, thioguanine, captopril, dimercaptopropanol, and dimercaptosuccinate. The quantified *K_i_* values were 18 (15), 35 (21), 46 (26), 16 (18), and 48 (37) µM for mercaptopurine, thioguanine, captopril, dimercaptopropanol, and dimercaptosuccinate, respectively, against the laccase of T. versicolor (A. oryzae) (Table 1 and Figure 3). To the best of our knowledge, these are the first organic small-molecule inhibitors of laccase. Meanwhile, sodium azide and AMTD exhibited *K_i_* values of 4 (3) and 12 (10) µM, respectively, against T. versicolor (A. oryzae). Both enzymes showed similar inhibition patterns and *K_i_* values for the seven inhibitors, which may reflect the closeness of their reaction mechanisms, despite the limited sequence identity. In addition, it is notable that 0.01% Triton X-100 was included in the enzyme-reaction mixture to avoid enzyme inhibition through colloidal aggregation effects by small molecules [16].

**Figure 4:**
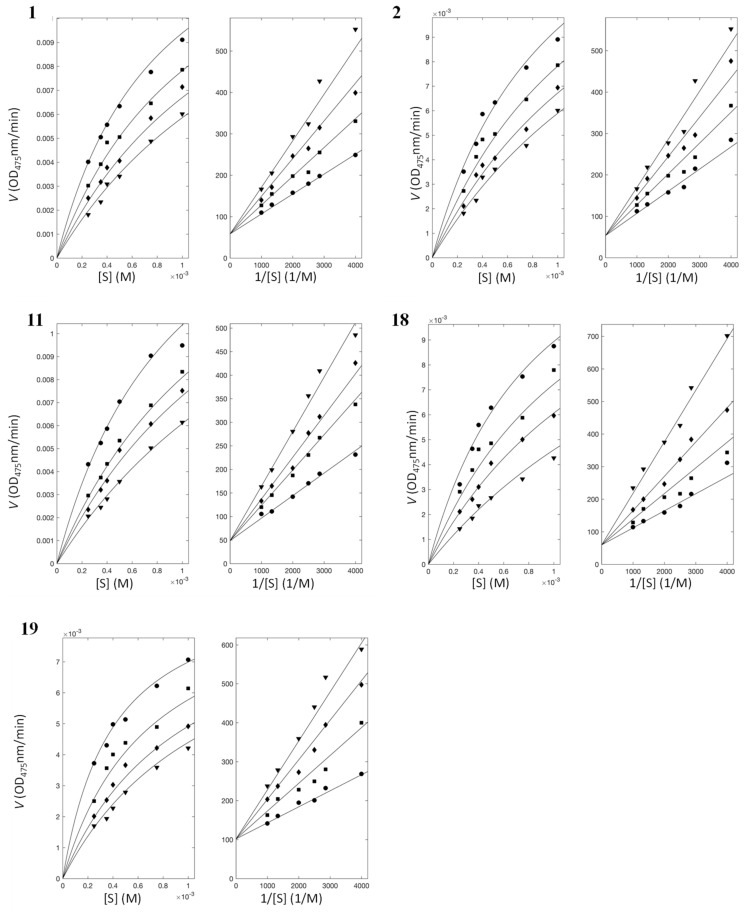
Enzyme inhibitory kinetics with *T. versicolor*. Left and right panels show Michaelis–Menten and Lineweaver–Burk plots, respectively, for each inhibitor: **1**, **2**, **11**, **18**, and **19**. ●, ■, ♦ and ▼ represent inhibitor concentrations of 10, 20, 30, and 40 µM for **1**; 15, 30, 45, and 60 µM for **2**; 30, 50, 60, and 80 µM for **11**; 10, 20, 30 and 50 µM for **18**; and 20, 45, 70, and 90 µM for **19**.

We also evaluated inhibitory enzyme kinetics using various concentrations of substrate and inhibitors. The nonlinear fittings of the enzyme kinetics classified all five new inhibitors, i.e., mercaptopurine, thioguanine, captopril, dimercaptopropanol, and dimercaptosuccinate, as competitive mode in the laccases from both sources (Figure 4 and Figure S7, and Table 2), indicating that the inhibitory mechanisms of all inhibitors are similar.

### 2.3. Differential Scanning Fluorimetry and Fluorescence-Quenching Experiments Support Direct Interactions between Small Molecules and Laccases

**Figure 5:**
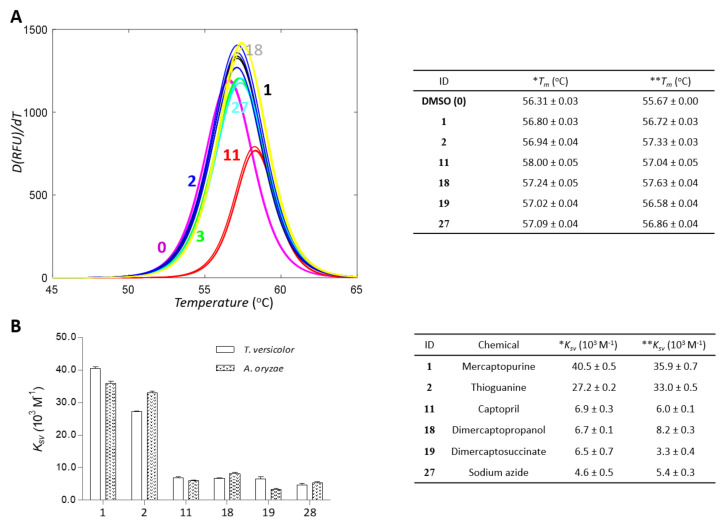
(**A**) Differential scanning fluorimetry (DSF) profiles of inhibitors with laccase from *T. versicolor*. DSF profiles for **0** (DMSO), **1** (mercaptopurine), **2 (**thioguanine), **11** (captopril), **18 (**mercaptopropanol), **19** (dimercaptosuccinate), and **27** (sodium azide), respectively. (**B**) Stern–Volmer constants obtained from the slope of the Stern–Volmer plot in fluorescence-quenching experiments are drawn and represented. * and ** represent laccase from *T. versicolor* and *A. oryzae*, respectively.

Differential scanning fluorimetry (DSF) confirmed direct binding of small molecules with laccases. Specific binding of a small molecule can either increase or decrease protein stability, resulting in a shift in melting temperature (T_m_) in DSF. All inhibitors increased the T_m_ values of laccases (Figure 5A and Figure S8). The changes were small but statistically significant for all the inhibitors (*p* < 0.005). Nevertheless, there was no apparent correlation between T_m_ and K_i_ values, likely indicating a complicated relationship in binding and inhibition by the small molecules.

The binding of small molecules can influence the surroundings of fluorophores and tryptophans in a protein, leading to the quenching of fluorescence signals. The altered patterns can support the direct interaction between a small molecule and a protein. We tested whether quenching of fluorescence signals from laccase occurred in a concentration-dependent manner with the inhibitors. The results clearly show that all inhibitors caused quenching (Figure 5B, Figure S9, and Figure S10). The decrements could be classified into two groups by values: one of mercaptopurine and thioguanine and the others of captopril, dimercaptopropanol, and mercaptopurine. The inhibitors in each group share chemical similarities in the functional moiety. This may support the reliability of the fluorescence-quenching results when interpreting the interaction between inhibitors and laccases. We employed two orthogonal methods, DSF and fluorescence quenching, and both supported direct binding.

### 2.4. Cheminformatics Analysis Revealed Differences between Tyrosinase and DBH Inhibitors and Suggested New Tyrosinase Inhibitors

One may argue that the test molecules in this study are biased from the viewpoint of chemical structures, thereby resulting in no finding of new DBH inhibitors. We performed a similarity ensemble approach (SEA) to determine the shared similarity between all tyrosinase inhibitors, the test molecules, and DBH inhibitors [37]. SEA reveals the distribution of pairwise Tanimoto coefficients (Tcs) between two small molecules from the respective groups. The distribution reflects the shared similarities of the inhibitors in the two groups.

**Figure 6:**
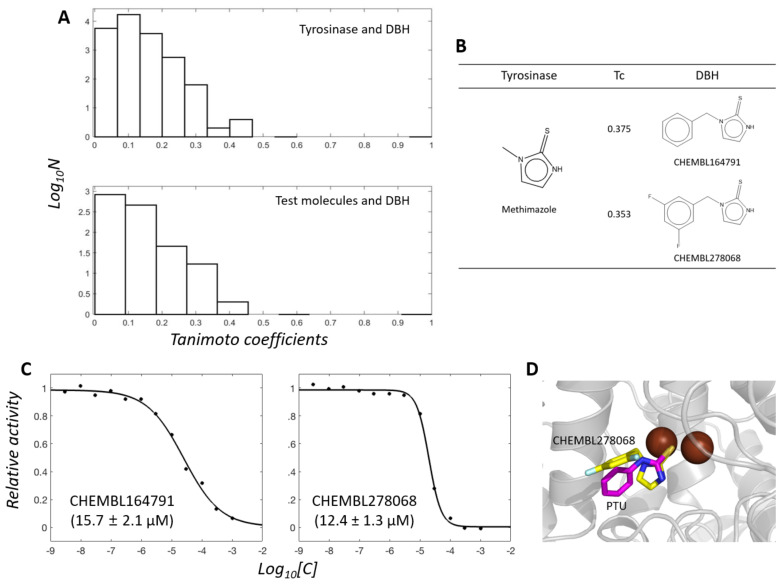
(**A**) Distribution of Tanimoto coefficients in the chemical pair from tyrosinase and DBH inhibitors. (**B**) Chemical similarity between methimazole and CHEMBL164791 and CHEMBL278068. (**C**) Inhibition profiles for tyrosinase by CHEMBL164791 and CHEMBL278068. Activities were scaled with relative values in the range of 0 to 1. Values in parentheses indicate *K_i_* values. (**D**) Docked pose of CHEMBL278068 (yellow) to tyrosinase. DOCK3.7 guided the docking of CHEMBL278068 into the mushroom tyrosinase coordinate (PDB code: 2Y9X). Brown spheres indicate copper atoms. The PTU structure (magenta) complexed with catechol oxidase [38] is overlaid for comparison.

We extracted 579 tyrosinase and 47 DBH organic inhibitors with molecular weights of <500 Da from the ChEMBL database. The 27,213 (579 × 47) Tcs between tyrosinase and DBH inhibitors share chemical similarities (Figure 6A). Tropolone is a dual inhibitor of tyrosinase and DBH. The pairs that include tropolone or its analogs showed the highest Tc values. The analogs are CHEMBL3357560 (Tc = 0.556 against tropolone), CHEMBL48310 (0.444), CHEMBL1275999 (0.440), CHEMBL1275969 (0.423), and CHEMBL135189 (0.409). Among them, DBH inhibitors include CHEMBL3357560 and CHEMBL135189. The others, CHEMBL48310, CHEMBL1275999, and CHEMBL1275969, belong to tyrosinase inhibitors. The close similarity to tropolone provoked us to postulate these analogs as dual inhibitors of tyrosinase and DBH. However, experimental validation was difficult to perform because of their commercial unavailability.

Interestingly, the 1222 (26 × 47) Tcs between the 26 tyrosinase inhibitors in this study (omitting two inhibitors, i.e., sodium azide and ATMD, from those in Figure 2) and the DBH inhibitors revealed a similar pattern of distribution. The resembled pattern qualitatively supports that the 26 test molecules can cover the chemical spaces that tyrosinase inhibitors map.

The second most highly ranked chemical moiety in pairs was thione. It is an MBG that is the second most common moiety in tyrosinase inhibitors, present in 202 (35%) of 579 ChEMBL-derived inhibitors. Our test molecules also included the 17 thione-containing molecules (Figure 2). Most DBH inhibitors are thione-containing molecules, accounting for 42 of 47 (89%) inhibitors. Of the test molecules, methimazole shared the highest Tc (0.375) with a DBH inhibitor, CHEMBL164791, among the thione-containing pairs (Figure 6B). Despite the apparent similarities with CHEMBL164791, neither methimazole nor other thione-containing test molecules significantly inhibited DBH at 50 µM. This implies that the additional aromatic part in CHEMBL164791 plays a substantial role in inhibition.

We then investigated whether CHEMBL164791 could inhibit tyrosinase activity. Both CHEMBL164791 and its analogous DBH inhibitor, CHEMBL278068 (Tc = 0.353 against methimazole), inhibited tyrosinase activity, with *K_i_* values of 15.7 and 12.4 µM, respectively (Figure 6C), which suggests that they are dual tyrosinase and DBH inhibitors. We also prepared the complex model between tyrosinase and new inhibitor CHEMBL278068 by docking simulation with the established protocol [14,15,16,17]. The model shows that CHEMBL278068 shares structural features with those in the complex between PTU and a plant catechol oxidase [38]. Their sulfur positions to the dicopper atoms were almost identical (Figure 6D). These data may suggest the idea that the presence of MBGs is sufficient for inhibition of tyrosinase, at least in thione-containing molecules. However, generalization of this idea will require further experimental evidence.

## 3. Discussion

How do the new inhibitors inhibit laccase? The results of fluorescence quenching and DSF can postulate that the inhibitions occur by direct interactions with laccase. Here, the complex structures of the newly found inhibitors with proteins may provide hints. Two crystal structures possess mercaptopurine as a ligand (PDB ligand name: PM6, https://www.rcsb.org/ligand/PM6 (accessed on 4 November 2021)). Their PDB codes are 3BGD and 3NS1. Thioguanine (ligand name: DX4) is found in four protein structures of 3JQA, 4M5M, 4XOY, and 4XP3. There are 14 protein structures containing captopril (ligand name: X8Z). Their PDB codes are 1J37, 2X8Z, 3LUS, 4C1D, 4C1F, 4C1H, 4C2P, 4DPR, 4EXS, 4PQA, 5AYA, 5ZIO, 6U10, and 6V61. Mercaptopurine and thioguanine are purine analogs and can inhibit purine-binding proteins. Captopril is a metalloenzyme-specific inhibitor. Therefore, dissection of the complex structures with captopril is informative. There are four types of metalloenzymes complexed with captopril: angiotensin-converting enzyme with mono-Zinc atoms (1J37, 2X8Z, 4C2P), metallo-β-lactamase with di-Zinc (4C1D, 4C1F, 4C1H, 4EXS, 5AYA, 5ZIO, 6U10, 6V61), leukotriene A4 hydrolase with mono-Zinc (4DPR), and desuccinylase with di-Zinc (4PQA). Figure S11 shows 2D diagrams of the intermolecular interaction between captopril and each type of metalloenzyme. The role of thiol moiety in captopril is to form chelating bonds to catalytic metals. The intermolecular distance between sulfur and metal is within 3 Å. It will be reasonable to assume that a similar mechanism exists in the inhibition of laccase by captopril. 

Where do the inhibitors bind to the laccase? The substrate binding site will be the primary candidate. We searched for known laccase structures deposited in the PDB database using the DALI server [39] and extracted 94 coordinates. They include three substrates in complex with laccase, which are 2,5-xylidine (PDB ligand: XYD and PDB code: 1KYA) [40], 4-methylbenzoic acid (4MA and 2HRG) [41], and sulfoacetate (AS8 and 2XYB). The molecules occupy identical positions on the laccase structures. The closest copper lies at the T1 site. However, the distances between the molecules and the T1 copper are greater than 6 Å. The direct chelation found in captopril and metalloenzyme complexes cannot be formed in this condition. We then searched for other potential sites where inhibitors can interact with copper atoms using FTMap [42] and p2rank [43] methods. However, there is no sufficient space near the copper clusters in the currently available structure (Figure S12). This may suggest the possibility of conformational fluctuation during the catalytic reaction. These complexities make it challenging to prepare an acceptable laccase-inhibitor docking model. Atomistic understanding of the underlying mechanism may necessitate the experimental 3D complex structure between laccase and inhibitor. 

No laccase inhibitor, except sodium azide, has been reported. Our study is one of the most extensive studies on laccase inhibitors. Because the physiological roles of laccases have been less studied in pathogens, therapeutic application of laccase inhibitors currently remains unclear. New inhibitors may trigger research in this direction. 

The cheminformatics-assisted approach to identify novel tyrosinase inhibitors in this study can be a meaningful example for finding new inhibitors. Several small molecules are known to inhibit various metalloenzymes [2,3]. For instance, captopril, a test molecule in this study, inhibits both zinc-containing angiotensin-converting enzymes and copper-containing tyrosinase. Systematic prediction and comparison across all metalloenzyme inhibitors and their MBGs may be the next step for widening the arsenal space of copper-containing enzyme inhibitors. It is noteworthy that the metal-binding pharmacophore library has shown its usefulness in drug discovery targeting metalloenzymes [1].

## 4. Materials and Methods

Cheminformatics and docking simulation —RDKit (Open-source cheminformatics; http://www.rdkit.org (accessed on 4 November 2021)) was used to calculate the pairwise Tcs between two molecules by employing ECFP4 as a molecular fingerprint. The data for inhibitors were extracted from ChEMBL25 [32,33]. Docking simulations were tried with DOCK 3.7 [44]. The procedures, in principle, followed what DOCK Blaster had previously described [45].

Enzyme activity with inhibitors—All chemical reagents were purchased from either Tokyo Chemical Industry (Tokyo, Japan), ChemBridge (San Diego, CA, USA), or Sigma-Aldrich (St. Louis, MO, USA). The reaction mixture for the tyrosinase assay comprised 20 nM tyrosinase and 500 µM L-DOPA in phosphate-buffered saline. The solution for the ceruloplasmin activity assay comprised 20 nM ceruloplasmin and 500 µM N,N-dimethyl-p-phenylenediamine (DMPD) in 100 mM of sodium acetate buffer at a pH of 5.5. The DBH activity assay was conducted in a reaction mixture containing 10 nM DBH, 500 µM DMPD, and 500 µM tyramine. The solution for the laccase activity assay comprised either 150 nM T. versicolor laccase or 350 nM A. oryzae laccase and 500 µM L-DOPA in 100 mM sodium phosphate buffer with a pH of 5.5. All the solutions included 0.01% Triton X-100 and 5% DMSO in the absence or presence of the inhibitor. After the addition of inhibitors, the solutions were incubated for 15 min at 30 °C. The absorbances of tyrosinase, laccase, ceruloplasmin, and DBH were measured at 475, 475, 550, and 515 nm, respectively, after the addition of substrate to the mixture solution, in a time-dependent manner. After confirming the molecules’ inhibitory activity at a single concentration of 50 µM, concentration-dependent inhibition was observed to calculate the IC_50_. The Cheng–Prusoff equation with *K_m_* was employed to convert the IC_50_ value into the inhibitory constant, *K_i_*, for each inhibitor [46].

Enzyme inhibitory kinetics—The velocities during the generation of the product for each enzyme were measured by varying the substrate and inhibitor concentrations. Simultaneous and nonlinear fitting of all profiles in an inhibitor minimized the Χ^2^ value of the differences of theoretical and experimental values in the Michaelis–Menten equation, expressed with the competitive, uncompetitive, noncompetitive, and mixed models. The fits resulted in the kinetics parameters *V_max_*, *K_m_*, *K_ic_*, and *K_iu_* in each model. The reduced Χ^2^ values between the models were compared based on F-statistic to select the most appropriate model [47]. All fittings and statistical analyses were conducted using MATLAB from MathWorks (Natick, MA, USA).

Differential scanning fluorimetry—DSF was performed to characterize the direct bindings of inhibitors to laccase. SYPRO^TM^ orange was added to the enzyme solution in the presence or absence of 500 µM inhibitor. The fluorescence of the dye was recorded at an excitation wavelength of 492 nm and an emission wavelength of 610 nm by increasing the temperature from 30°C to 85°C. The RT-PCR CFX96 system from BioRad (Hercules, CA, USA) detected the time- and temperature-dependent signals. The mid-point melting temperature (Tm) was determined by nonlinearly minimizing the following equation.

(1) $$I\left(T\right)=LL+\frac{UL-LL}{\left[1+\exp(\frac{Tm-T}{a})\right]}$$

where *LL* and *UL* indicate the top and baseline of the curves, respectively, and *a* indicates the steepness of the slope. Signals in the range of 45 °C to 65 °C were used for the fitting.

Fluorescence quenching—Fluorescence quenching was performed to evaluate the direct interaction between the laccases and their inhibitors. The changes in fluorescence intensity of laccase with excitation (λ_ex_) at 280 nm and emission (λ_em_) at 310 nm were recorded under various inhibitor concentrations. The concentration of laccase was 60 nM, and those for ligands were 0, 0.003, 0.01, 0.03, 0.1, 0.3, 1, 3, 10, 30, 100, and 300 µM. All spectrophotometric data in this study were obtained using Synergy neo2 from BioTek (Winooski, VT, USA). Changes in the fluorescence intensity by the addition of ligands with the concentration [*L*] were interpreted according to the Stern–Volmer equation:

(2) $$\frac{F0}{F}=1+Kq\tau 0\left[L\right]=1+Ksv\;\left[L\right]$$

where *F* and *F0* are the fluorescence intensity of enzymes with and without inhibitors, respectively. *K_q_* is the quencher rate constant, and τ_0_ is the lifetime of emissive excited state of the enzyme without ligands. *K_sv_* is the Stern–Volmer quenching constant. All experiments in this study were conducted in triplicate, independently, to generate mean and uncertainty values. 

## Figures and Tables

**Table 1:** Quantified inhibitory constants (*K_i_*) of the tested compounds against DBH, ceruloplasmin, and laccases from *T. versicolor* and *A. oryzae*.

ID.	Chemical	DBH	Ceruloplasmin	*T. versicolor*	*A. oryzae*
**1**	Mercaptopurine	−	−	18.3 ± 2.4	15.1 ± 4.6
**2**	Thioguanine	−	−	35.3 ± 4.6	21.2 ±1.7
**11**	Captopril	−	−	46.7 ± 3.2	26.3 ± 2.4
**12**	Kojic acid	−	−	>1000	>1000
**18**	Dimercaptopropanol	57.9 ± 7.6	−	16.1 ± 1.5	18.6 ± 3.3
**19**	Dimercaptosuccinate	−	−	48.1 ± 1.8	37.3 ± 5.6
**24**	PTU	−	−	>1000	>1000
**26**	Tropolone	9.3 ± 5.5	−	>1000	>1000
**27**	Sodium azide	70.6 ± 11.6	1.6 ± 0.3	4.2 ± 1.3	3.1 ± 1.7
**28**	ATMD	23.7 ± 2.7	10.9 ± 1.4	12.3 ± 1.3	10.5 ± 2.5

All inhibitory constant values are in μM.

**Table 2:** Parameters of inhibitory kinetics for laccases.

ID	Chemical	Mechanism	* *K_i_* (µM)	** *K_i_* (µM)
**1**	Mercaptopurine	Competitive	2.4	17.2
**2**	Thioguanine	Competitive	23.4	26.2
**11**	Captopril	Competitive	5.2	32.4
**18**	Dimercaptopropanol	Competitive	9.5	18.0
**19**	Dimercaptosucciniate	Competitive	14.1	13.1

* and ** represent the simulated inhibitory constants by nonlinear fitting, assuming competitive inhibition in *T. versicolor* and *A. oryzae*, respectively.

## Supplementary Materials

The supplementary materials are available online at https://www.mdpi.com/article/10.3390/ijms222413661/s1.

## References

[1] Cohen S.M. (2017). A bioinorganic approach to fragment-based drug discovery targeting metalloenzymes. Acc. Chem. Res..

[2] Day J.A., Cohen S.M. (2013). Investigating the selectivity of metalloenzyme inhibitors. J. Med. Chem..

[3] Chen A.Y., Adamek R.N., Dick B.L., Credille C.V., Morrison C.N., Cohen S.M. (2019). Targeting metalloenzymes for therapeutic intervention. Chem. Rev..

[4] Weekley C.M., He C. (2017). Developing drugs targeting transition metal homeostasis. Curr. Opin. Chem. Biol..

[5] Riccardi L., Genna V., De Vivo M. (2018). Metal—ligand interactions in drug design. Nat. Rev. Chem..

[6] Caspi R., Billington R., Keseler I.M., Kothari A., Krummenacker M., Midford P.E., Ong W.K., Paley S., Subhraveti P., Karp P.D. (2020). The MetaCyc database of metabolic pathways and enzymes—A 2019 update. Nucleic Acids Res..

[7] Valasatava Y., Rosato A., Furnham N., Thornton J.M., Andreini C. (2018). To what extent do structural changes in catalytic metal sites affect enzyme function?. J. Inorg. Biochem..

[8] Bowman S.E., Bridwell-Rabb J., Drennan C.L. (2016). Metalloprotein crystallography: More than a structure. Acc. Chem. Res..

[9] Zolghadri S., Bahrami A., Hassan Khan M.T., Munoz-Munoz J., Garcia-Molina F., Garcia-Canovas F., Saboury A.A. (2019). A comprehensive review on tyrosinase inhibitors. J. Enzyme Inhib. Med. Chem..

[10] Pillaiyar T., Manickam M., Namasivayam V. (2017). Skin whitening agents: Medicinal chemistry perspective of tyrosinase inhibitors. J. Enzyme Inhib. Med. Chem..

[11] Beliaev A., Learmonth D.A., Soares-da-Silva P. (2006). Synthesis and biological evaluation of novel, peripherally selective chromanyl imidazolethione-based inhibitors of dopamine β-hydroxylase. J. Med. Chem..

[12] McCarthy J.R., Matthews D.P., Broersma R.J., McDermott R.D., Kastner P.R., Hornsperger J.M., Demeter D.A., Weintraub H.J., Whitten J.P. (1990). 1-(Thienylalkyl) imidazole-2 (3H)-thiones as potent competitive inhibitors of dopamine. beta.-hydroxylase. J. Med. Chem..

[13] Kruse L.I., Kaiser C., DeWolf W.E., Frazee J.S., Erickson R.W., Ezekiel M., Ohlstein E.H., Ruffolo R.R., Berkowitz B.A. (1986). Substituted 1-benzylimidazole-2-thiols as potent and orally active inhibitors of dopamine. Beta.-hydroxylase. J. Med. Chem..

[14] Choi J., Choi K.-E., Park S.J., Kim S.Y., Jee J.-G. (2016). Modeling. Ensemble-based virtual screening led to the discovery of new classes of potent tyrosinase inhibitors. J. Chem. Inf. Model.

[15] Choi J., Jee J.-G. (2015). Repositioning of thiourea-containing drugs as tyrosinase inhibitors. Int. J. Mol. Sci..

[16] Choi J., Lee Y.-M., Jee J.-G. (2018). Thiopurine drugs repositioned as tyrosinase inhibitors. Int. J. Mol. Sci..

[17] Choi J., Park S.-J., Jee J.-G. (2015). Analogues of ethionamide, a drug used for multidrug-resistant tuberculosis, exhibit potent inhibition of tyrosinase. Eur. J Med. Chem..

[18] Saghaie L., Pourfarzam M., Fassihi A., Sartippour B. (2013). Synthesis and tyrosinase inhibitory properties of some novel derivatives of kojic acid. Res. Pharm. Sci..

[19] Sambasiva Rao K., Tripathy N., Srinivasa Rao D., Prakasham R. (2013). Production, characterization, catalytic and inhibitory activities of tyrosinase. Res. J. Biotechnol..

[20] Bento I., Peixoto C., Zaitsev V.N., Lindley P.F. (2007). Ceruloplasmin revisited: Structural and functional roles of various metal cation-binding sites. Acta Crystallogr. Sect. D Biol. Crystallogr..

[21] Hellman N., Gitlin J. (2002). Ceruloplasmin metabolism and function. Annu. Rev. Nutr..

[22] Han I.W., Jang J.-Y., Kwon W., Park T., Kim Y., Lee K.B., Kim S.-W. (2017). Ceruloplasmin as a prognostic marker in patients with bile duct cancer. Oncotarget.

[23] Roberti M.D.R.F., Borges Filho H.M., Gonçalves C.H., Lima F.L. (2011). Aceruloplasminemia: A rare disease-diagnosis and treatment of two cases. Rev. Bras. Hematol. Hemoter..

[24] Rahman M.K., Rahman F., Rahman T., Kato T. (2009). Dopamine-β-hydroxylase (DBH), its cofactors and other biochemical parameters in the serum of neurological patients in Bangladesh. Int. J. Biomed. Sci..

[25] Vendelboe T.V., Harris P., Zhao Y., Walter T.S., Harlos K., El Omari K., Christensen H.E. (2016). The crystal structure of human dopamine β-hydroxylase at 2.9 Å resolution. Sci. Adv..

[26] Tishchenko K., Beloglazkina E., Mazhuga A., Zyk N. (2016). Copper-containing enzymes: Site types and low-molecular-weight model compounds. Rev. J. Chem..

[27] Strong P., Claus H. (2011). Laccase: A review of its past and its future in bioremediation. Cri. Rev. Environ. Sci. Technol..

[28] Martínez-Sotres C., Rutiaga-Quiñones J.G., Herrera-Bucio R., Gallo M., López-Albarrán P., Technology (2015). Molecular docking insights into the inhibition of laccase activity by medicarpin. Wood Sci. Technol.

[29] Wang T., Xiang Y., Liu X., Chen W., Hu Y. (2017). A novel fluorimetric method for laccase activities measurement using Amplex Red as substrate. Talanta.

[30] Shiro Y. (2012). Structure and function of bacterial nitric oxide reductases: Nitric oxide reductase, anaerobic enzymes. Biochim. Biophys. Acta. Bioenerg..

[31] Jumper J., Evans R., Pritzel A., Green T., Figurnov M., Ronneberger O., Tunyasuvunakool K., Bates R., Zidek A., Potapenko A. (2021). Highly accurate protein structure prediction with AlphaFold. Nature.

[32] Mendez D., Gaulton A., Bento A.P., Chambers J., De Veij M., Félix E., Magariños M.P., Mosquera J.F., Mutowo P., Nowotka M. (2019). ChEMBL: Towards direct deposition of bioassay data. Nucleic Acids Res..

[33] Davies M., Nowotka M., Papadatos G., Dedman N., Gaulton A., Atkinson F., Bellis L., Overington J.P. (2015). ChEMBL web services: Streamlining access to drug discovery data and utilities. Nucleic Acids Res..

[34] Kuo T., Ho F. (2013). Competitive inhibition of mushroom tyrosinase by captopril. Res. J. Biotechnol..

[35] Espín J.C., Wichers H.J. (2001). Effect of captopril on mushroom tyrosinase activity in vitro. Biochim. Biophys. Acta.

[36] Meck C., D’Erasmo M.P., Hirsch D.R., Murelli R.P. (2014). The biology and synthesis of α-hydroxytropolones. Med. Chem. Comm..

[37] Keiser M.J., Roth B.L., Armbruster B.N., Ernsberger P., Irwin J.J., Shoichet B.K. (2007). Relating protein pharmacology by ligand chemistry. Nat. Biotechnol..

[38] Klabunde T., Eicken C., Sacchettini J.C., Krebs B. (1998). Crystal structure of a plant catechol oxidase containing a dicopper center. Nat. Struct. Biol..

[39] Holm L., Rosenstrom P. (2010). Dali server: Conservation mapping in 3D. Nucleic Acids Res..

[40] Bertrand T., Jolivalt C., Briozzo P., Caminade E., Joly N., Madzak C., Mougin C. (2002). Crystal structure of a four-copper laccase complexed with an arylamine: Insights into substrate recognition and correlation with kinetics. Biochemistry.

[41] Matera I., Gullotto A., Tilli S., Ferraroni M., Scozzafava A., Briganti F. (2008). Crystal structure of the blue multicopper oxidase from the white-rot fungus Trametes trogii complexed with p-toluate. Inorganica Chim. Acta.

[42] Kozakov D., Grove L.E., Hall D.R., Bohnuud T., Mottarella S.E., Luo L., Xia B., Beglov D., Vajda S. (2015). The FTMap family of web servers for determining and characterizing ligand-binding hot spots of proteins. Nat. Protoc..

[43] Krivak R., Hoksza D. (2018). P2Rank: Machine learning based tool for rapid and accurate prediction of ligand binding sites from protein structure. J. Cheminformatics.

[44] Bender B.J., Gahbauer S., Luttens A., Lyu J., Webb C.M., Stein R.M., Fink E.A., Balius T.E., Carlsson J., Irwin J.J. (2021). A practical guide to large-scale docking. Nat. Protoc..

[45] Irwin J.J., Shoichet B.K., Mysinger M.M., Huang N., Colizzi F., Wassam P., Cao Y. (2009). Automated docking screens: A feasibility study. J. Med. Chem..

[46] Yung-Chi C., Prusoff W.H. (1973). Relationship between the inhibition constant (KI) and the concentration of inhibitor which causes 50 per cent inhibition (I50) of an enzymatic reaction. Biochem. Pharmacol..

[47] Dias A.A., Pinto P.A., Fraga I., Bezerra R.M. (2014). Diagnosis of enzyme inhibition using Excel Solver: A combined dry and wet laboratory exercise. J. Chem. Educ..

