# Supplementary material for: New Inhibitors of Laccase and Tyrosinase by Examination of Cross-Inhibition between Copper-Containing Enzymes
Source: Int J Mol Sci. 2021 Dec 20;22(24):13661. doi: 10.3390/ijms222413661 (PMC8707586; doi:10.3390/ijms222413661)
Supplement: Supplementary file 1 [file ijms-22-13661-s001.zip › ijms-1472838-supplementary.pdf]

Supplementary materials

New Inhibitors of Laccase and Tyrosinase by Examination of Cross-Inhibition between Copper-containing Enzymes

Dinesh Chaudhary, Fangchen Chong, Trilok Neupane, Joonhyeok Choi and Jun-Goo Jee

A

	T. versicolor	*A. oryzae*	Ceruloplasmin	DBH	Tyrosinase
T. versicolor	1.0	0.866	0.687	0.294	0.289
A. oryzae	0.727	1.0	0.589	0.273	0.247
Ceruloplasmin	0.352	0.358	1.0	0.208	0.169
DBH	0.273	0.290	0.337	1.0	0.204
Tyrosinase	0.344	0.337	0.353	0.260	1.0

B

	T. versicolor	*A. oryzae*	Ceruloplasmin	DBH	Tyrosinase
T. versicolor		0.268	0.167	0.047	0.047
A. oryzae	2.38		0.151	0.043	0.040
Ceruloplasmin	4.05	4.46		0.060	0.050
DBH	7.13	7.39	7.59		0.038
Tyrosinase	7.33	7.22	7.35	7.49	

Figure S1. Similarities of sequences and structures of copper-containing enzymes. (A) TM-scores between copper-containing enzymes. TM-score has values in the range of 0 to 1. The higher value indicates the closer overall similarity. TM-scores are normalized by the length of proteins listed in the leftmost column. **(B)** RMSD (root mean square deviation) values of two aligned protein structures (left side of diagonal) and the portion of the identical amino acids in the two aligned protein sequences (right side of diagonal). *T. versicolor* and *A. oryzae* mean the species names that contain the laccases in this study. The PDB codes of the 3D structures are 1GYC, 4ENZ, 4ZEL, and 2Y9X for *T. versicolor*, ceruloplasmin, DBH, and tyrosinase, respectively. The structure of *A. oryzae* was prepared using Alphafold2 (Figure 1). The respective lengths of sequences for *T. versicolor*, *A. oryzae*, ceruloplasmin, DBH, and tyrosinase are 499, 598, 1030, 550, and 391. The RMSD values have the unit of Å.

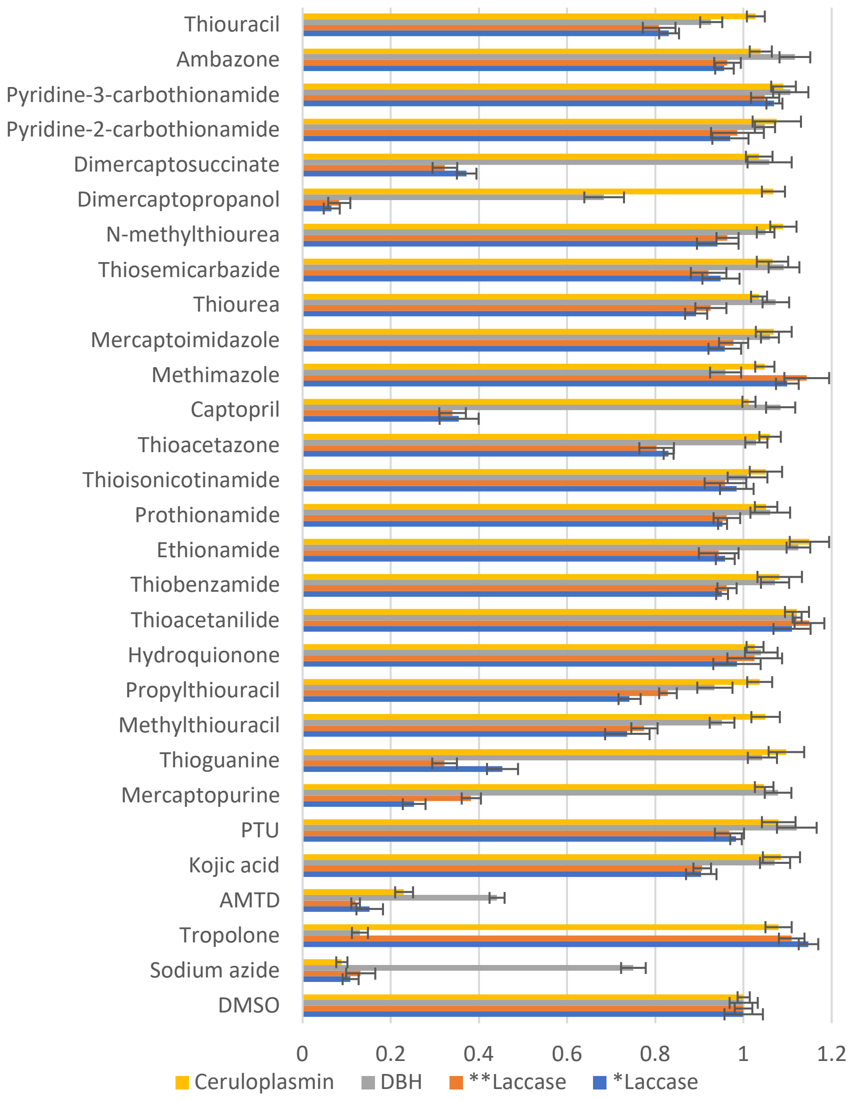

Figure S2. Screening of small molecules against copper-containing enzymes; Enzyme activities measured with *laccase (*T. versicolor*), **laccase (*A. oryzae*), dopamine-β-hydroxylase (DBH), and ceruloplasmin at a concentration of 50 μM are expressed with the relative activity compared to that treated with DMSO.

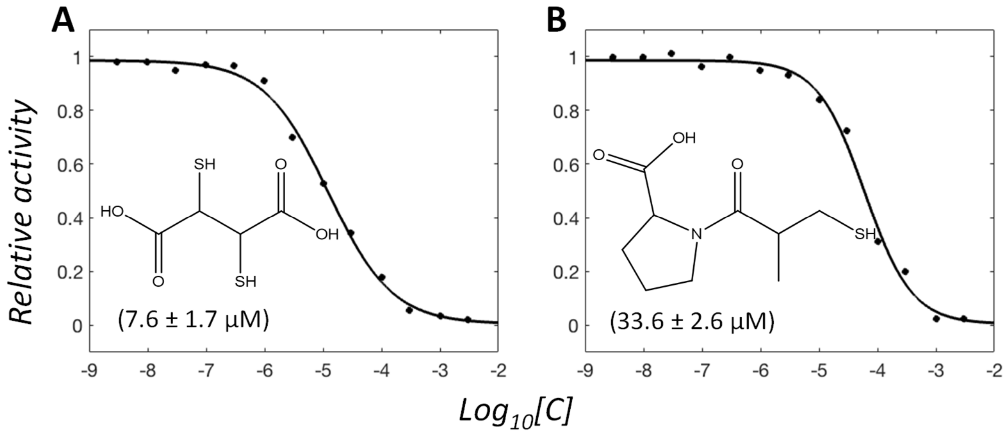

Figure S3. Graph showing the concentration-dependent inhibition of mushroom tyrosinase exhibited by **(A)** dimercaptosuccinate and **(B)** captopril. The activity was normalized from 0 to 1. The value in the figure represents the inhibitory constant (K_i).

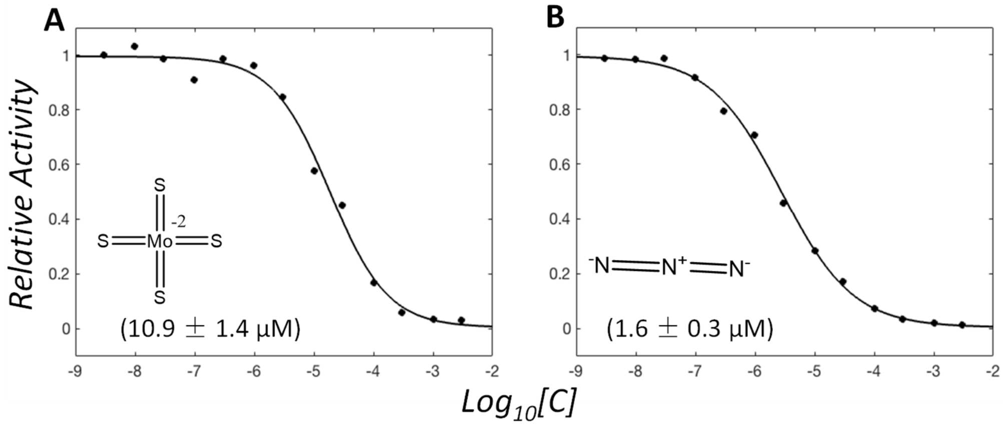

Figure S4. Graphs showing the concentration-dependent inhibition of ceruloplasmin by inorganic molecules: (**A)** ATMD, and (**B)** sodium azide. The activities were scaled to have values between 0 and 1. The value in the figure represents the inhibitory constant (K_i).

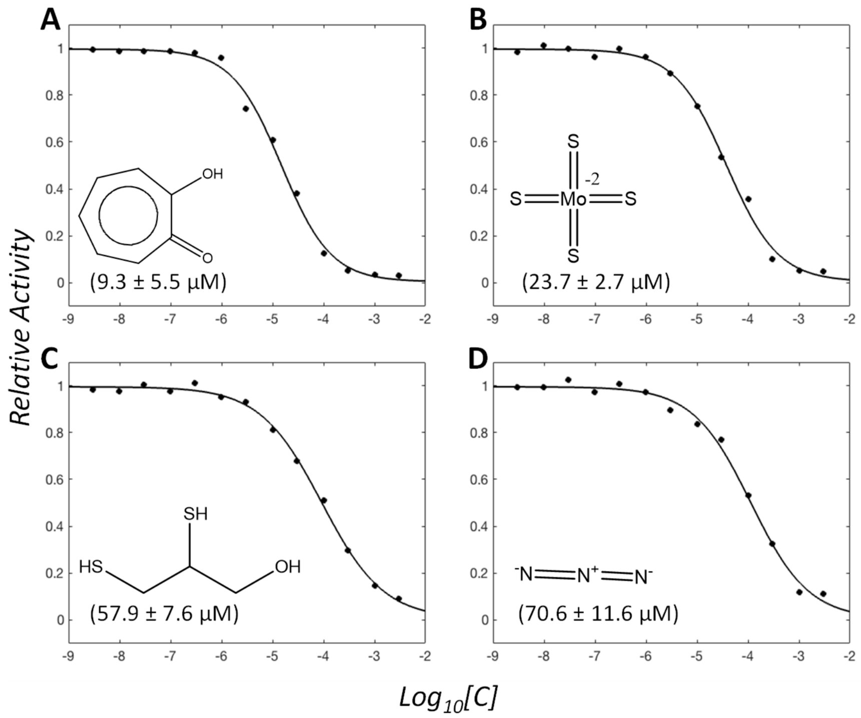

Figure S5. Graph showing the concentration-dependent inhibition of DBH by **(A)** tropolone, **(B)** ATMD, **(C)** dimercaptopropanol, and **(D)** sodium azide. The activities were scaled to a relative value in the range of 0 to 1. The value in the figure represents the inhibitory constant (K_i).

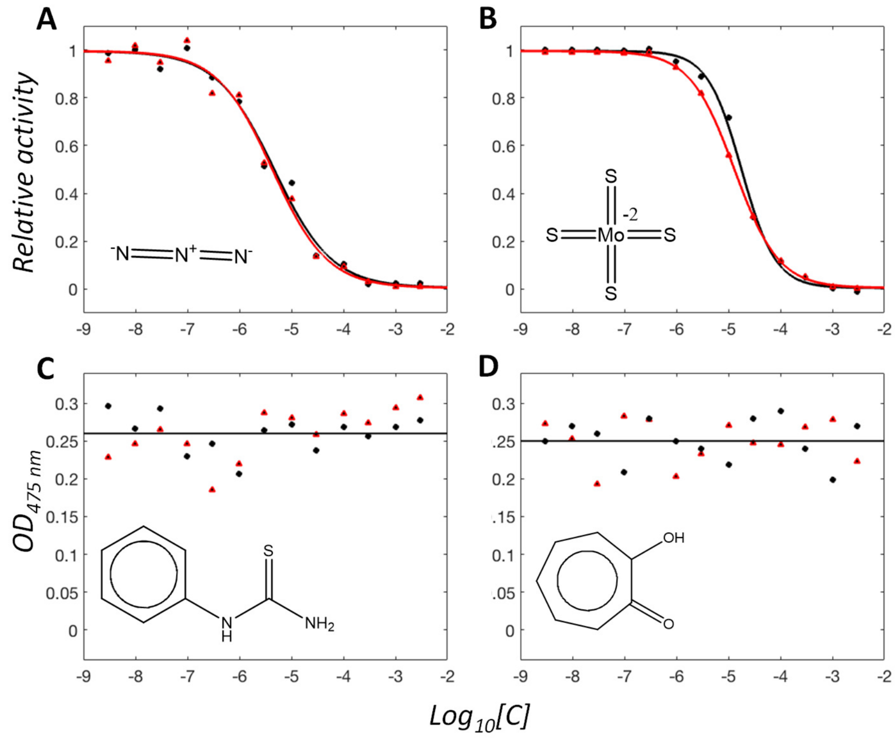

Figure S6. Graph showing the concentration-dependent profiles of **(A)** sodium azide (reference inhibitor) and **(B)** ATMD against laccase from *T. versicolor* (black dot) and *A. oryzae* (red dot). The activity is scaled from 0 to 1. Optical densities of **(C)** PTU, and **(D)** tropolone are shown at a wavelength of 475 nm (OD_{475}).

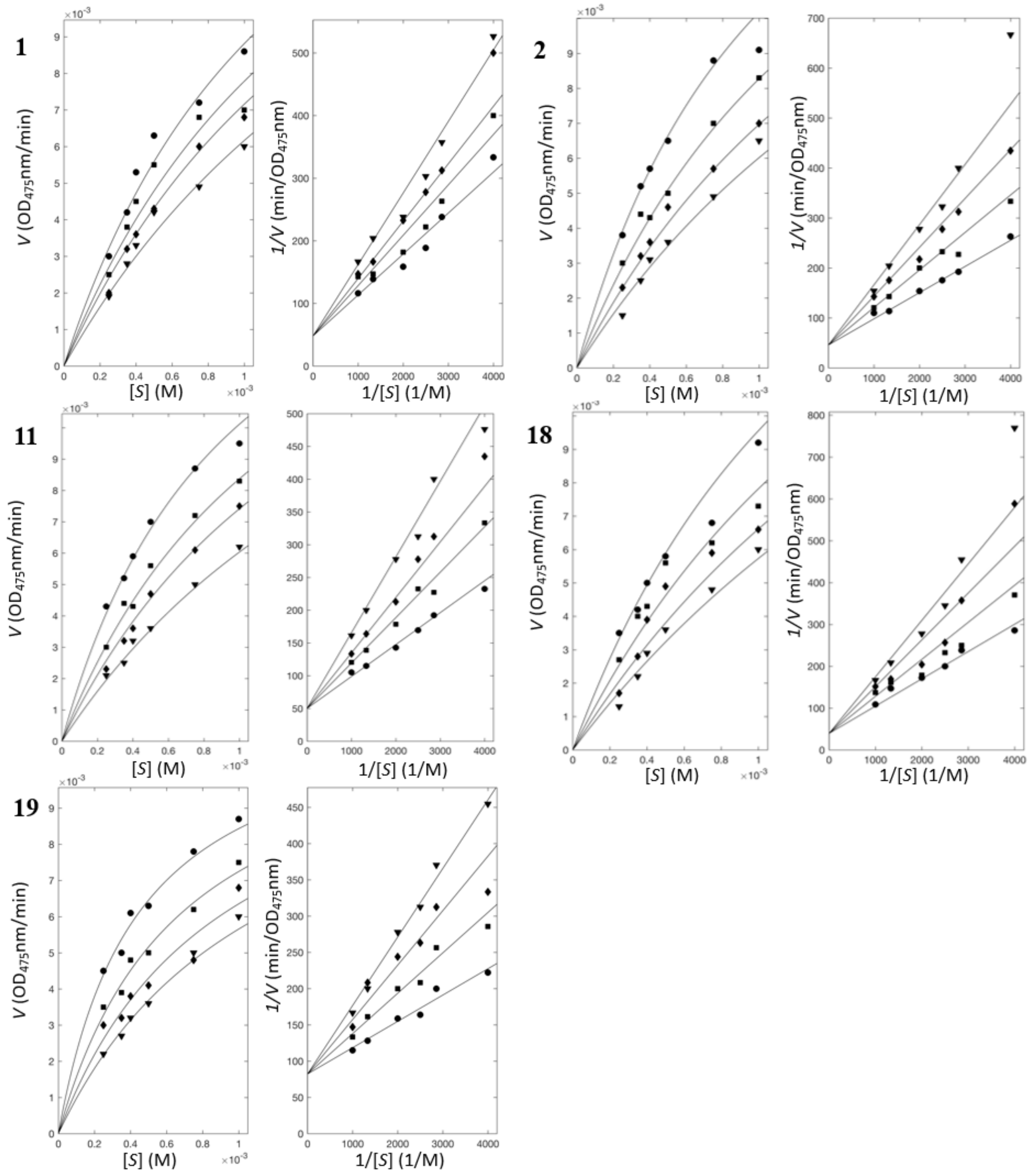

Figure S7. Enzyme inhibitor kinetics with laccase (*A. oryzae*) inhibitors. The left and right panels show the Michaelis-Menten and Lineweaver-Burk plots, respectively for each compound; **1 (**mercaptopurine), **2** (thioguanine),**11** (captopril), **18** (dimercaptopropanol), and **19** (dimercaptosuccinate). ●, ■, ◆, and ▼ represent inhibitor concentrations of 10, 15, 20 and 30 μM for **1**; 20, 40, 60, and 80 μM for **2**; 15, 35, 50, and 80 for **11**, 10, 20, 40 and 60 μM for **18**; and 15, 30, 45, and 60 μM for **19**.

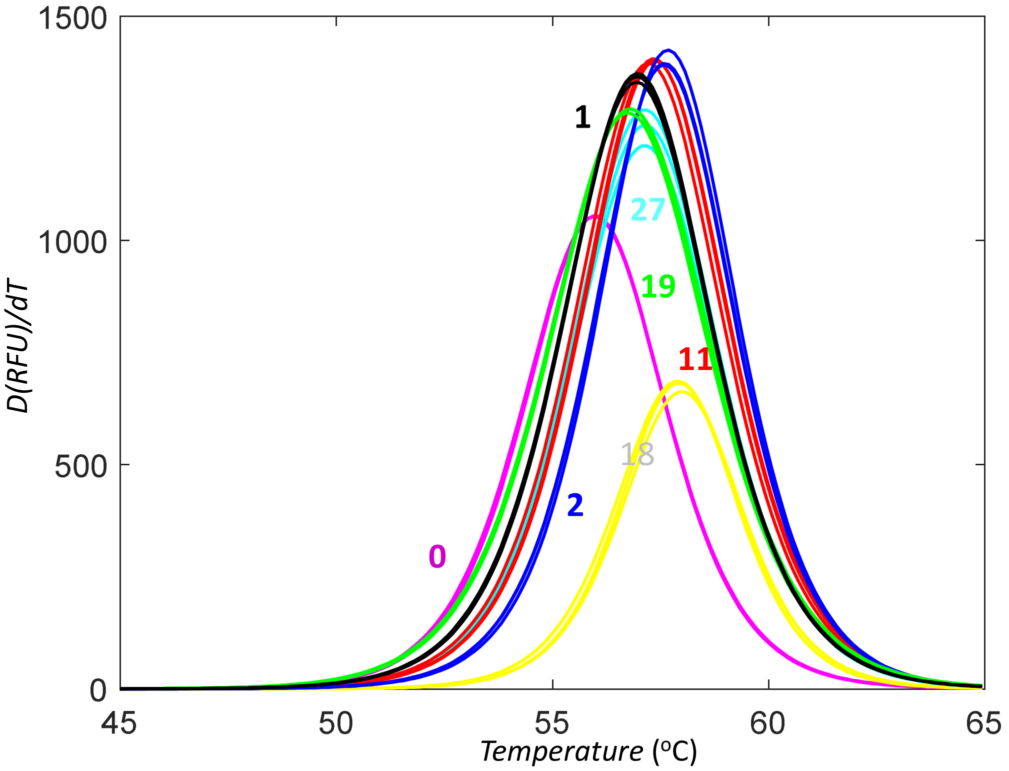

Figure S8. Profiles of differential scanning fluorimetry (DSF) analyses of inhibitors with laccase from *A. oryzae*. DSF for temperature are plotted as magneta, black, blue, red, yellow, green, and cyan for **0** **(**DMSO, enzyme only), **1 (**mercaptopurine), **2 (**thioguanine), **11** (captopril), **18 (**mercaptopropanol), **19** **(**dimercaptosuccinate), and **27** (sodium azide), respectively.

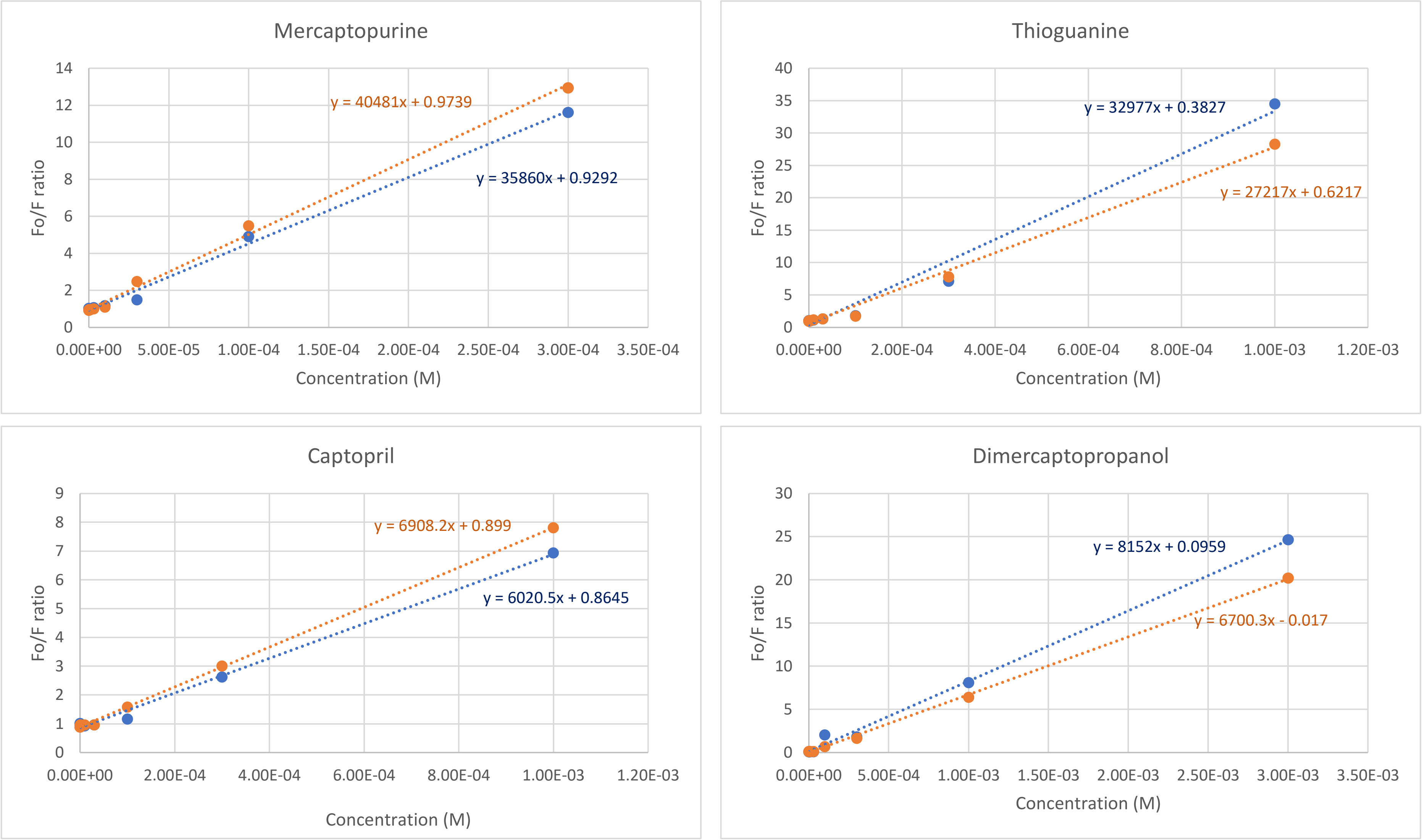
Mercaptopurine
y = 40481x + 0.9739
y = 35860x + 0.9292
Fo/F ratio
Concentration (M)
Thioguanine
y = 32977x + 0.3827
y = 27217x + 0.6217
Fo/F ratio
Concentration (M)
Captopril
y = 6908.2x + 0.899
y = 6020.5x + 0.8645
Fo/F ratio
Concentration (M)
Dimercaptopropanol
y = 8152x + 0.0959
y = 6700.3x - 0.017
Fo/F ratio
Concentration (M)

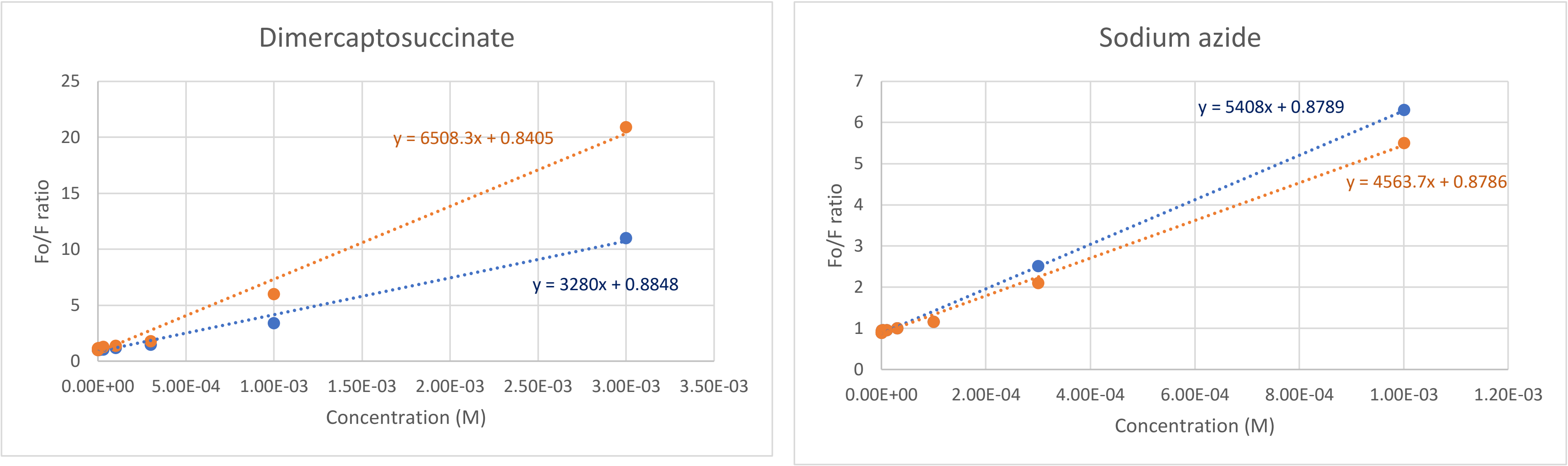

Figure S9. Stern–Volmer constant of laccase from *T. versicolor* (orange dot line) and *A*. oryzae (blue dot line) inhibitors represented with equation calculated from the concentration-dependent responses in fluorescence emission of laccase.

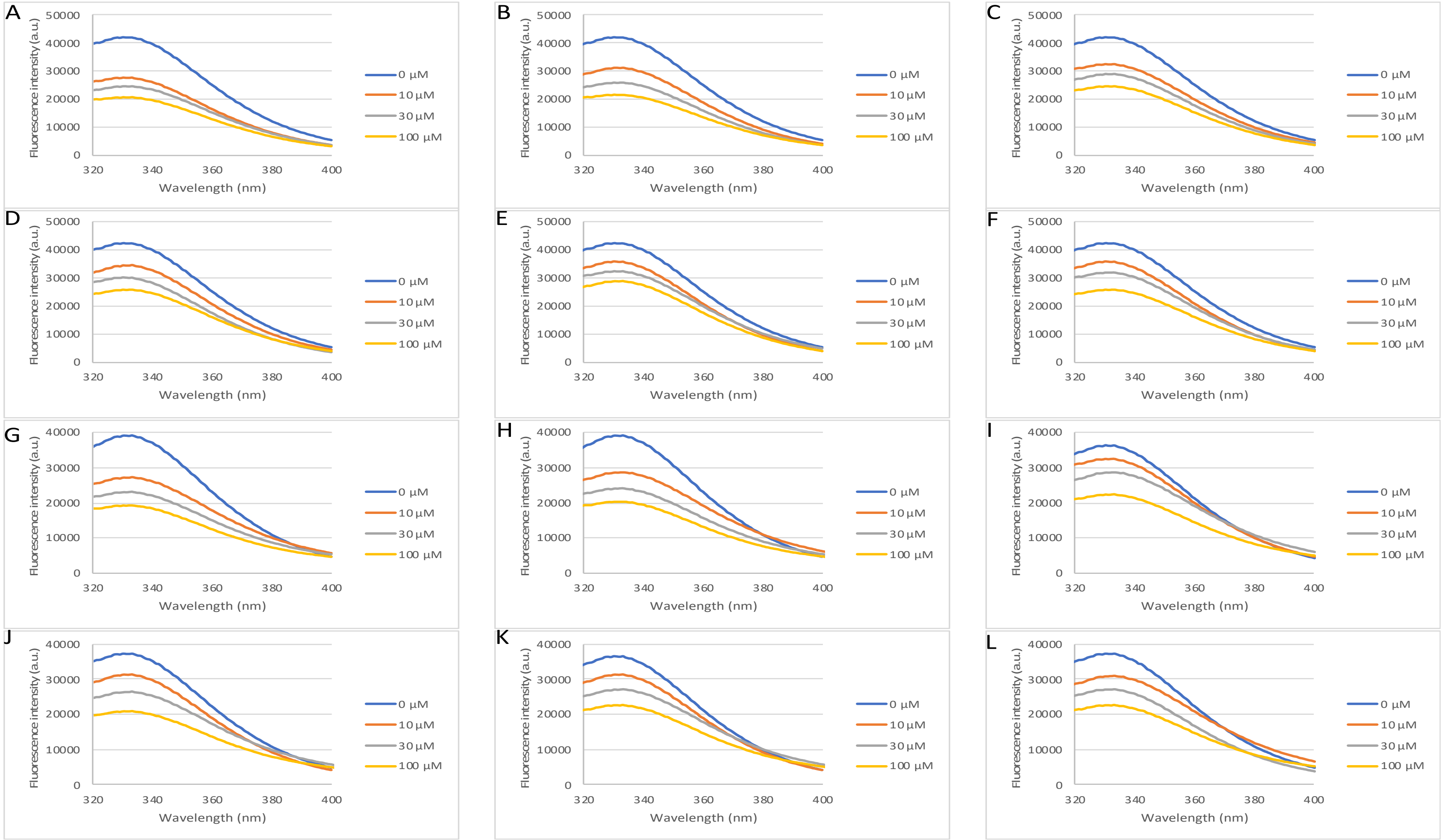

Figure S10. Concentration-dependent fluorescence emission quenching of laccase from *T. versicolor* **(A-F)** and *A. oryzae* **(G-L)**. The profiles of the ligands mercaptopurine **(A, G)**, thioguanine **(B, H)**, captopril **(C, I)**, dimercaptopropanol **(D, J)**, dimercaptosuccinate **(E, K)**, and sodium azide **(F, L)** are represented at concentrations 0, 10, 30, and 100 µM.

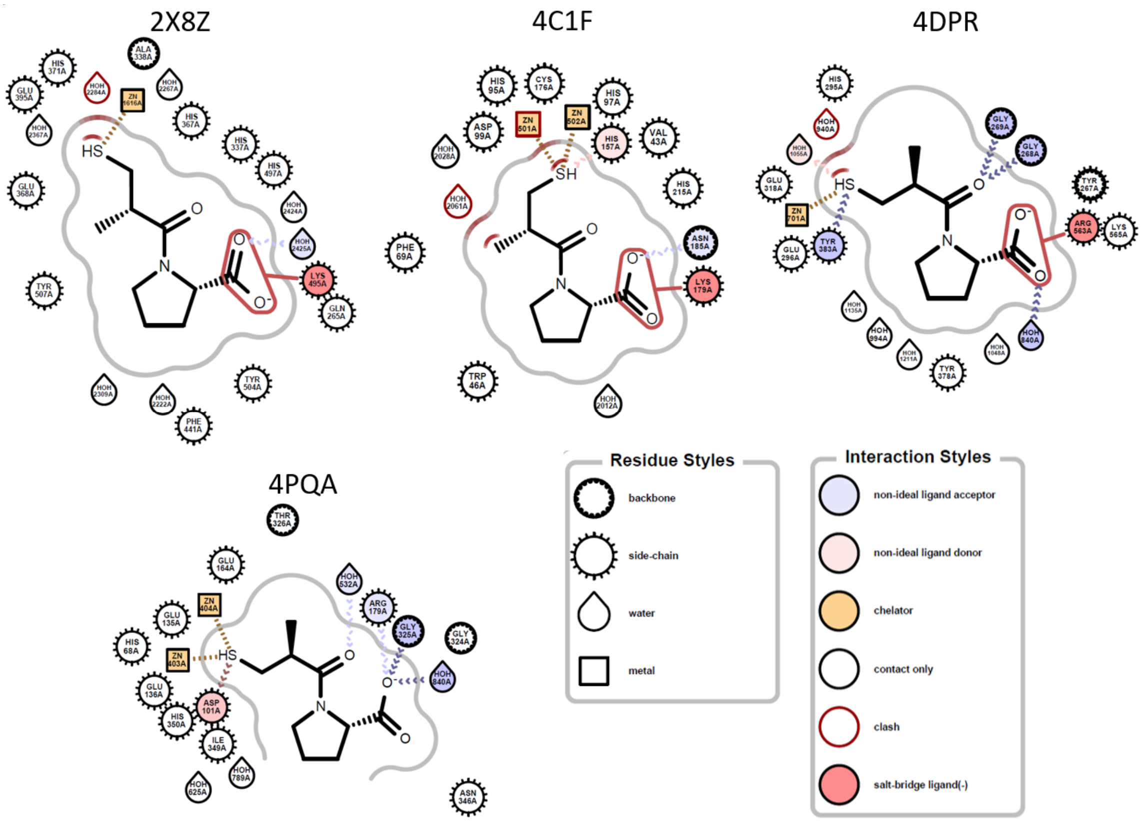

Figure S11. 2D diagrams of intermolecular interaction between captopril and complexed metalloenzymes. The PDB codes for the corresponding complex are labeled. The diagrams are prepared using Openeye Grapheme Toolkit. The residue and interaction styles in the diagrams are written as well. The proteins of 2X8Z, 4C1F, 4DPR, and 4PQA correspond to angiotensin-converting enzyme (mono-Zinc), metallo-β-lactamase (di-Zinc), leukotriene A4 hydrolase (mono-Zinc), and desuccinylase (di-Zinc), respectively.

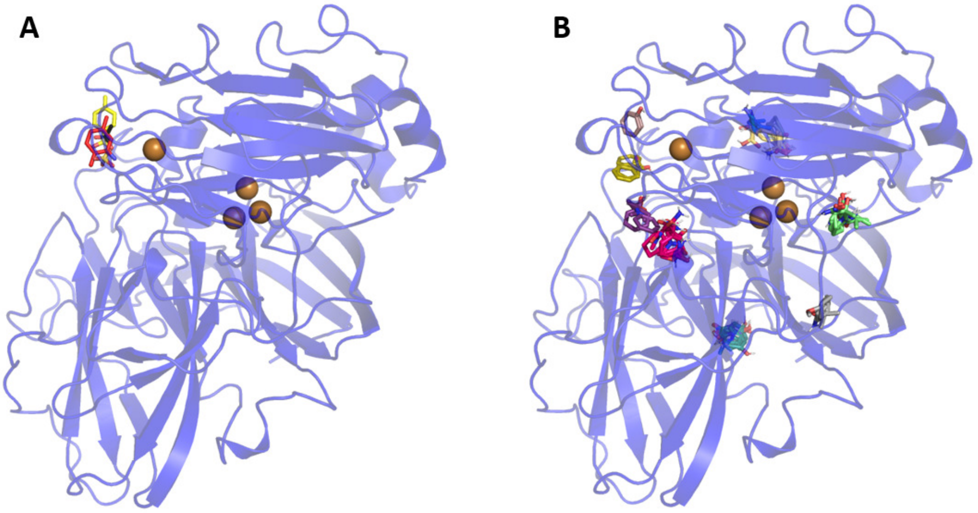

Figure S12. Laccase structures with substrates and putative small molecule binding sites. (A) Laccase structure (PDB code: 1GYC) with substrates, 2,5-xylidine, 4-methylbenzoic acid, and sulfo acetate, are presented. Three substrates are extracted from PDBs of 1KYA, 2HRG, and 2XYB, respectively. **(B)** FTMap-derived putative small molecule binding sites are prepared with the corresponding fragments. Two structures (A & B) are aligned to have an identical direction for comparison.
